# Genome-Wide Expression Analysis of Long Noncoding RNAs and Their Target Genes in Metafemale *Drosophila*

**DOI:** 10.3390/ijms24098381

**Published:** 2023-05-06

**Authors:** Xinyu Liu, Ran Yan, Haosheng Liu, Shuai Zhang, Ruixue Wang, Bowen Zhang, Lin Sun

**Affiliations:** 1 Beijing Key Laboratory of Gene Resource and Molecular Development, College of Life Sciences, Beijing Normal University, Beijing 100875, China; 2 State Key Laboratory of Earth Surface Process and Resource Ecology, College of Life Sciences, Beijing Normal University, Beijing 100875, China; 3 Ministry of Education Key Laboratory for Biodiversity Science and Ecological Engineering, College of Life Sciences, Beijing Normal University, Beijing 100875, China; 4 Key Laboratory of Cell Proliferation and Regulation Biology of Ministry of Education, College of Life Science, Beijing Normal University, Beijing 100875, China

**Keywords:** aneuploidy, lncRNA, dosage compensation, inverse dosage effect, genomic imbalance

## Abstract

Aneuploidy is usually more detrimental than altered ploidy of the entire set of chromosomes. To explore the regulatory mechanism of gene expression in aneuploidy, we analyzed the transcriptome sequencing data of metafemale *Drosophila*. The results showed that most genes on the X chromosome undergo dosage compensation, while the genes on the autosomal chromosomes mainly present inverse dosage effects. Furthermore, long noncoding RNAs (lncRNAs) have been identified as key regulators of gene expression, and they are more sensitive to dosage changes than mRNAs. We analyzed differentially expressed mRNAs (DEGs) and differentially expressed lncRNAs (DELs) in metafemale *Drosophila* and performed functional enrichment analyses of DEGs and the target genes of DELs, and we found that they are involved in several important biological processes. By constructing lncRNA-mRNA interaction networks and calculating the maximal clique centrality (MCC) value of each node in the network, we also identified two key candidate lncRNAs (CR43940 and CR42765), and two of their target genes, *Sin3A* and *MED1*, were identified as inverse dosage modulators. These results suggest that lncRNAs play an important role in the regulation of genomic imbalances. This study may deepen the understanding of the gene expression regulatory mechanisms in aneuploidy from the perspective of lncRNAs.

## 1. Introduction

Variations in the number of chromosomes can generally be divided into two categories: euploid variation and aneuploid variation [1,2]. The former refers to the increase or decrease in the entire chromosome complement, which results in the production of polyploids or haploids [2]. The latter usually manifests as the addition or loss of individual chromosomes or chromosome segments from a diploid [1]. Both variations have clear impacts on the survival and development of different organisms [3,4,5], such as yeast [6], maize [7] and *Arabidopsis* [8]. However, the impact of aneuploidy, caused by a phenomenon referred to as “genomic imbalance”, on an individual is usually more severe than that of altered ploidy of a whole set of chromosomes [9]. Multiple previous studies have confirmed this viewpoint [9,10,11,12]. In addition, aneuploidy is also commonly associated with some serious diseases in humans. Trisomy of chromosome 21 usually results in a series of clinical features, such as short stature, decreased neuronal density, cerebellar hypoplasia and intellectual disability, a constellation of phenotypes commonly referred to as Down syndrome (DS) [13]. Aneuploidy of sex chromosomes can also lead to Crane syndrome (47, XXX) and Turner syndrome (45, X) [14]. Furthermore, aneuploidy is generally considered to be one of the main markers of tumors. According to statistical analysis, approximately 90% of solid tumors have aneuploidy characteristics [1,15]. Therefore, the study of aneuploidy can help explain a variety of biological phenomena and enhance the understanding of cancer and other diseases.

Early studies on gene expression in aneuploid organisms at the mRNA or protein level showed that genes on chromosomes with dosage changes generally exhibit a dosage effect, that is, the expression level of a gene is positively correlated with its dosage [4,16]. Dosage effect was proved by deletion and repetition of *ry+* in *Drosophila melanogaster* [17]. However, many subsequent studies have also shown that the expression of a considerable number of genes on the chromosomes with dosage changes is the same as that in normal diploids, i.e., dosage compensation occurs [18,19,20,21,22]. In *Drosophila*, gene expression in males with a single copy of the X chromosome is upregulated, to a degree roughly matching that of the double copies of the X chromosome in females [23]. The male-specific lethal (MSL) complex, the protein product of the *Drosophila* male-specific lethal gene, has been regarded as a classical model to explain the dosage compensation effect in *Drosophila*. This complex is specifically enriched on the male X chromosome and is involved in mediating the upregulation of genes on the male X chromosome to 200% that in diploid female *Drosophila*. More interestingly, it was found that the X-linked genes in metafemale (XXX; AA) *Drosophila*, with the absence of the MSL complex, were also dosage compensated when compared with those in normal diploid females (XX; AA), and similar results were identified in male *mle/mle* mutants without the complex [24,25]. Therefore, further investigation into the expression pattern and regulatory mechanism in metafemales should be conducted in the future [26].

Additionally, a study of the trisomy of autosomal chromosomes of *Drosophila* showed that the genes on the trisomic chromosomes also exhibited dosage compensation, while the expression of genes on the other unchanged chromosomes was negatively correlated with the dosage in the changed regions, a phenomenon called the inverse dosage effect [20,21,27,28]. For example, regarding trisomy of the 2L chromosome of *Drosophila*, the expression of most genes on the 2L chromosome is the same as that in normal diploid individuals, while the expression of genes on other chromosomes is reduced to two-thirds of that in normal diploid individuals [29]. Gene expression analysis of maize and *Arabidopsis* further confirmed this phenomenon [8,18,19,30]. Based on this, dosage compensation can be interpreted as the result of the inverse dosage effect counteracting the positive dosage effect of the gene whose dosage changes [21]. The discovery of autosomal dosage compensation also suggests that, in addition to the MSL complex, there may be broader mechanisms regulating dosage compensation and inverse dosage effects in aneuploid cells.

Studies of the regulators of white eye color reporter genes in *Drosophila* showed that the regulatory mechanism of aneuploid dosage change can be narrowed down to the role of a single gene [31,32], such as *inverse regulator-a* (*Inr-a*). The molecular functions of these genes were studied, and it was found that most of the modifier genes encode transcription factors, chromatin modification proteins and signal transduction molecules [33]. These modifier genes are dosage dependent, and this regulation is likely to be mediated through a concentration-dependent cascade. In addition, a common feature of these dosage-sensitive modifier genes is that they tend to be components of macromolecular complexes [34].

Based on the above studies, scientists proposed the gene balance hypothesis (GBH) to explain the mechanism of genomic imbalance. The theory holds that stoichiometric changes in the components of multisubunit complexes affect the assembly kinetics of the complexes, which in turn affects the number of functional products and gene expression patterns, ultimately affecting the phenotype and fitness of individuals [5]. Although many complex events are involved in this process, with the development of bioinformatics and transcriptome sequencing technologies, genome-wide expression analyses of aneuploidy, such as studies in *Drosophila* and maize, are constantly confirming this hypothesis.

Long noncoding RNAs (lncRNAs) have been determined to be widely expressed in cells and play an important role in the regulation of gene expression [35]; lncRNAs are defined as RNA molecules with more than 200 nt and no protein-coding capacity. The difference between lncRNAs and mRNAs is that the former have fewer exons than the latter, and the expression abundance and stability of the former in different tissues are lower [36]. In view of their high heterogeneity, lncRNAs are most commonly classified based on their position in the genome relative to protein-coding genes and include intergenic lncRNAs, intronic lncRNAs, enhancer RNAs (eRNAs), bidirectional lncRNAs and antisense lncRNAs [37].

Due to the flexibility of RNA transcripts and their ability to fold into complex 3D conformations, lncRNAs can participate in specific interactions with nucleic acid and protein molecules through complementary base pairing and structure recognition, respectively. Therefore, a single lncRNA molecule can interact with a variety of macromolecules. This also shows that lncRNAs have great potential for involvement in many biological functions [36]. lncRNAs are involved in maintaining the structural integrity of the nucleus and can regulate the expression of nearby genes (in the nucleus, acting in cis) or the expression of distant genes (in the nucleus or cytoplasm, acting in trans) by interacting with proteins, RNA and DNA [38]. In eukaryotes, the regulation of gene expression is quite complex and compartmentalized. It can occur in multiple steps, for example, structural modification of chromatin, recruitment of the transcription machinery, mRNA processing and delivery to the cytoplasm, mRNA degradation, mRNA translation and post-translational processes, all of which can be affected by lncRNAs [37]. For example, in the structural modification of chromatin, the lncRNA Xist can mediate the inactivation of one X chromosome in female mammals by recruiting PRC2, a histone methyltransferase; in addition, in the dosage compensation of the X chromosome in male *Drosophila*, roX1 and roX2 extend in cis on the X chromosome and recruit other MSL complex components for H4K16 acetylation [37]. lncRNAs also play key roles in the alternative splicing and degradation of mRNA [39,40]. In addition, recent studies have shown that lncRNAs are also associated with cell differentiation, organogenesis, tissue homeostasis and pathological conditions, such as cancer and cardiovascular diseases [41,42]. In a single-cell sequencing study of oocytes, fertilized eggs and cells after the first cell division in early mouse embryos, lncRNA transcripts were identified at various stages of detection, and certain lncRNAs were expressed at specific developmental stages [43]. With the increasing research on lncRNAs, lncRNAs have also become recognized as important biomarkers and drug targets under certain pathological conditions.

Transcription factors (TFs) are a class of proteins regulating gene expression, leading to transcription initiation by binding to regulatory DNA sequences in cell genomes and recruiting RNA polymerases and cofactors to target genes [44]. TFs are vital to organisms because they play important roles in signal transduction and gene regulation [45]. TFs can be activated by either intrinsic or extrinsic signals; the ultimate result of TF activation is the regulation of expression of its direct target genes [45]. The combined activity of a set of TFs connected to their targeted genes is known as a gene regulatory network (GRN) [46]. However, the exact nature of GRNs is unknown, and understanding of the impact of TFs on transcriptome changes is lacking [45]. Moreover, it has been found that the influence of aneuploidy can be reduced to the action of single genes in *Drosophila* and that the genes that respond to dosage effects of aneuploidy usually include TFs, signal transduction components and chromatin proteins [33,34]. Therefore, we conducted genome-wide analysis of TFs in differentially expressed mRNAs (DEGs) and the target genes of differentially expressed lncRNAs (DELs).

Changes in the stoichiometry of macromolecular complexes mediate the response of aneuploid gene expression patterns to dosage changes, but the specific molecular mechanisms and regulatory networks involved in this process are still unclear. In this study, global differentially expressed lncRNAs between metafemales and normal diploid females were identified through transcriptome sequencing and bioinformatics methods, and a differential lncRNA-mRNA interaction network was constructed to screen key regulatory genes in the network. This study attempts to reveal certain key regulators in the hierarchical regulatory network related to aneuploid genomic imbalance from the perspective of lncRNAs and to deeply understand the molecular mechanism of gene expression regulation of aneuploidy.

## 2. Results

### 2.1. Distribution of Gene Expression Ratios in Metafemale Individuals

**Figure 1:**
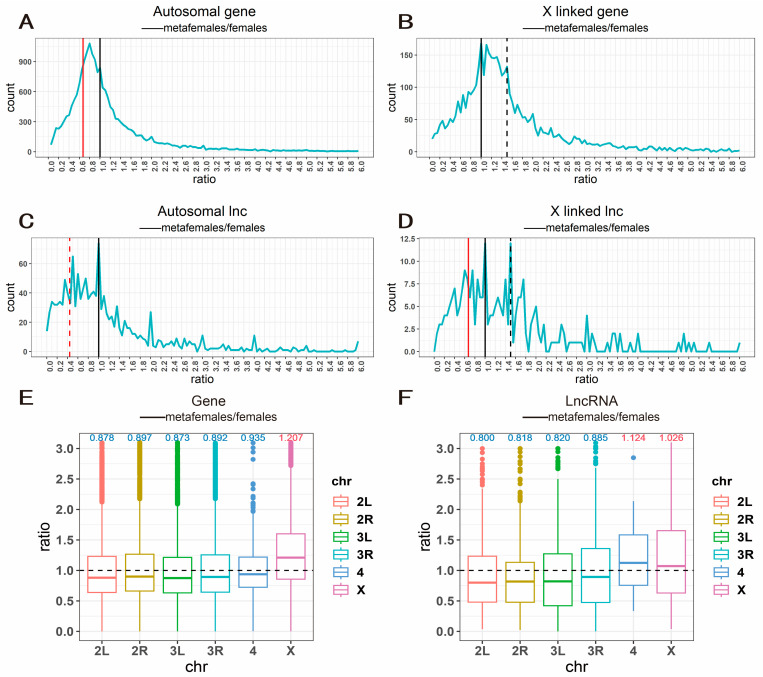
Ratio distribution of gene expression of metafemale flies. When mapping, it is divided into autosomes and X chromosomes according to the location of the transcripts on the chromosome and divided into mRNAs and lncRNAs according to the type of transcripts. (**A**,**B**) Ratio distributions of gene expression in metafemale (XXX) compared with normal diploid individuals. All genes are divided into autosomal genes (**A**) and X linked genes (**B**) according to their positions on chromosomes. The vertical red solid line represents the ratio 0.67 (the ratio of inverse dosage effects (2/3)), the vertical black solid line represents the ratio 1.00 (no change) and the vertical black dashed line shows the ratio 1.50 (the ratio of gene dosage effects (3/2)). The frequencies were plotted in bins of 0.05. (**C**,**D**) Ratio distribution of lncRNAs in metafemale (XXX) compared with normal diploid individuals. All genes are divided into autosomal lncRNAs (**C**) and X linked lncRNAs (**D**) according to their positions on chromosomes. The vertical red solid line represents 0.67 (the ratio of inverse dosage effects (2/3)), the vertical black solid line represents 1.00 (no change), the vertical red dashed line represents 0.44 (the ratio of double inverse dosage effect (0.67 × 0.67)) and the vertical black dashed line represents 1.5 (the ratio of gene dosage effects (3/2)). The horizontal axis represents the ratio of gene expression values compared to normal females, and the vertical axis represents the frequency ratios that fall into each bin of 0.05. (**E**,**F**) Boxplots of gene expression ratios of mRNAs (**E**) and lncRNAs (**F**) on individual chromosomes; the top numbers represent the medians of gene expression ratios, where red indicates upregulation and blue indicates downregulation.

To verify the dosage compensation and inverse dosage effect phenomenon in aneuploid *Drosophila* and to explore the changes in the expression levels of lncRNAs during this process, we used normal diploid female *Drosophila* as a control to plot the expression ratio frequency distribution curves of mRNAs and lncRNAs of metafemale *Drosophila*. The changes in mRNA expression levels in metafemale *Drosophila* were similar to those previously reported. For autosomes, the peak ratio of the frequency distribution of mRNAs was mainly concentrated between 0.67 (inverse dosage effect) and 1.0 (no change) (Figure 1A). Although this value is not numerically inversely proportional to the dosage change on the X chromosome (1.5), the leftward shift of the peak still indicates that most genes on the autosome are regulated by the inverse dosage effect. The corresponding boxplot also showed a similar trend (Figure 1E). The dosage change of genes on the X chromosome was 1.5-fold, while the expression level was centered around a ratio of 1.0 (Figure 1B), indicating that dosage compensation occurred for most genes on the X chromosome. The expression ratios of genes on the X chromosome were slightly increased due to the change in dosage but were less than 1.5, indicating that dosage compensation occurred for most genes, while the expression levels of genes on other chromosomes were decreased, indicating that they were regulated by the inverse dosage effect. In addition, we were interested in the effects of aneuploidy on mitochondrial genes; therefore, we also plotted the expression ratio frequency distribution curve of mitochondrial DNA of metafemale *Drosophila* (Figure S1A). We found that the ratio of the frequency distribution of mitochondrial DNA was centered around 1.5, suggesting that mitochondrial genes were regulated by the dosage effect. Additionally, there was a shoulder peak near a ratio of 0.44 (0.67 × 0.67), indicating that certain genes were regulated by a double inverse dosage effect [21,29,34]. These results show that the influence of XXX on mitochondrial genes is extensive.

Through high-throughput sequencing, we obtained the genome-wide expression levels of lncRNAs in metafemale *Drosophila* for the first time and plotted the frequency distribution curves of lncRNAs on autosomes and sex chromosomes in metafemale *Drosophila* by the method described above (Figure 1C,D). Through comparative analysis, it was found that in *Drosophila*, lncRNAs on both autosomes and the X chromosome were more sensitive to changes in chromosome dosage than mRNAs. In metafemales, lncRNAs on autosomal chromosomes were generally downregulated compared with those in normal diploid females, and the gene expression levels were mainly concentrated in the range of 0.44–1.0 and were centered around ratios of 0.44 (red dashed line, double inverse dosage effect, 0.67 × 0.67) and 1.0 (black line, no change). The expression levels of lncRNAs on the X chromosome were also concentrated in a wide range (0.67–1.5), indicating that the lncRNAs on the X chromosome were significantly affected by dosage changes, exhibiting both a dosage effect and an inverse dosage effect, i.e., excessive dosage compensation. In the corresponding boxplot (Figure 1F), the changes in lncRNA expression were similar to those in mRNA expression, with slight upregulation of lncRNAs on chromosome X and downregulation of lncRNAs on autosomes (except for chromosome 4), and the median expression levels indicated that the magnitude of change in lncRNA expression was larger than that in mRNA expression (Figure 1E,F), which further suggested that lncRNAs are more sensitive to changes in sex chromosome dosage. In addition, it is worth noting that the median expression level of lncRNAs on chromosome 4, unlike the other autosomes, was higher than 1.0, a phenomenon that may be related to its different evolutionary paths and heterochromatin composition [34].

### 2.2. Identification of Differentially Expressed mRNAs and lncRNAs

**Figure 2:**
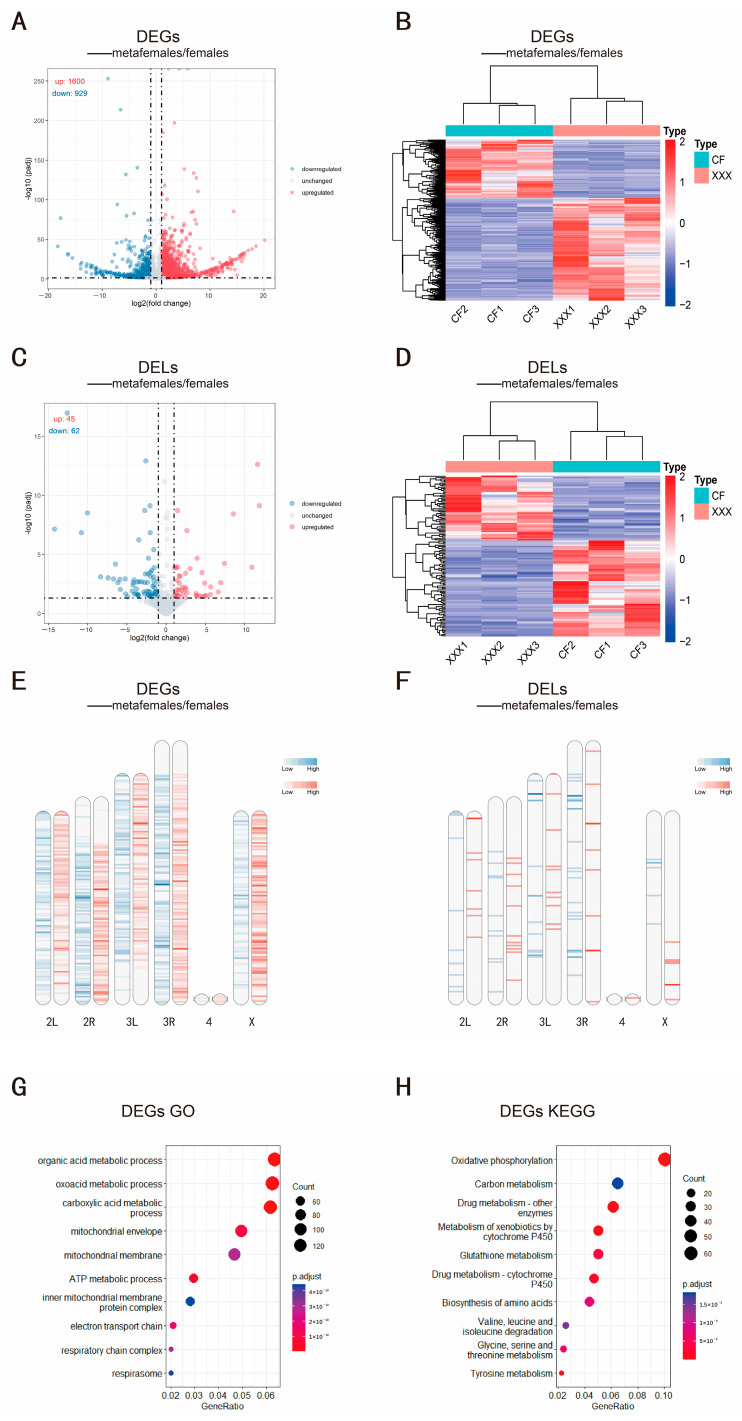
DEG and DEL analysis of metafemale and diploid females and enrichment of DEGs. (**A**,**B**) Volcano map and heatmap of DEGs, where CF represents normal diploid female *Drosophila* and XXX represents metafemale *Drosophila*. (**C**,**D**) Volcano map and heatmap of DELs. (**E**,**F**) Density heatmaps of the distribution of DEGs and DELs along chromosomes in metafemale *Drosophila*. The red bands represent upregulated DEGs or DELs, and the blue bands represent downregulated DEGs or DELs, respectively. The darker colors show a greater density of the DEGs or DELs. (**G**) Bubble chart of GO enrichment analysis of differential genes. (**H**) Bubble chart of KEGG enrichment analysis of differential genes. The picture shows the top 10 terms of the enrichment results, where the *X*-axis represents the gene ratio, that is, the ratio of the number of genes in a certain term to the number of genes in all alignments, and the *y*-axis represents the specific enriched term. The color of the bubble reflects the *p*-adjust value of the enrichment, and the size of the bubble reflects the number of genes enriched in the term.

To DEGs and DELs in metafemale *Drosophila*, differential expression analysis was performed on the expression matrix using the R package DESeq2. The results showed that there were 2529 DEGs (|log2FC| > 1, padj < 0.05) (Figure 2A,B) and 107 DELs (|log2FC| > 1, padj < 0.05) (Figure 2C,D) in metafemale flies compared with normal diploid female flies. Among DEGs, 1600 were upregulated and 929 were downregulated, i.e., most were upregulated (Figure 2A). DELs, however, showed the opposite trend, with 45 upregulated and 62 downregulated (Figure 2C). We classified the DELs based on their position in the genome relative to protein-coding genes (Figure S2A). According to the pie chart, it is obvious that LincRNAs account for the majority of the DELs (56.07%), followed by intronic lncRNAs (34.58%), and that the numbers of antisense lncRNAs and sense lncRNAs were relatively small, accounting for 5.61% and 3.74% of the DELs, respectively. The heatmap shows that metafemale *Drosophila* can be well distinguished from normal diploid female *Drosophila* through these DEGs and DELs (Figure 2B,D). In addition, by observing the density distribution of the DEGs and DELs on the chromosomes, it was found that the upregulated and downregulated genes were widely distributed across the chromosomes (Figure 2E,F), suggesting that aneuploidy has a wide range of effects on gene expression across the whole genome, and this broad effect was observed for both mRNAs and lncRNAs. However, there are more downregulated lncRNAs than mRNAs in metafemale *Drosophila*, indicating that lncRNAs may be more sensitive to changes in chromosome dosage and are regulated by a more obvious inverse dosage effect. We also analyzed the differential expression of mitochondrial genes (Figure S1B), but the results were not significant because the number of differentially expressed mtDNAs was too small.

To understand the impact of aneuploidy on *Drosophila*, GO functional and KEGG pathway enrichment analyses were performed on the DEGs between metafemale *Drosophila* and normal diploid female *Drosophila* (Figure 2G,H). The GO enrichment analysis results showed that these DEGs were mainly related to the metabolic processing of various types of organic matter, the mitochondrial energy production process, material transmembrane transport, the regulation of anatomical structure and morphology and organ development, the response to extracellular stimuli, learning and memory, etc., and the KEGG pathway enrichment analysis results showed that the differentially expressed genes were involved in oxidative phosphorylation, metabolic pathways of various organic substances and biosynthesis of insect hormones. We focused on the influence of aneuploidy on genes that encode transcription factors; to this end, we identified the transcription factors enriched among the DEGs. By enrichment analysis of TF-binding motifs in the sequences 5 kb upstream of the DEGs in metafemale *Drosophila*, regulatory transcription factors with high confidence were selected through determination of these overrepresented motifs. Motifs were screened according to the normalized enrichment score (NES) > 3, and the 10 binding motifs with the highest scores are listed in Figure S3. By annotation with highconf annotation, 25 transcription factors enriched among the upregulated DEGs and 13 transcription factors enriched among the downregulated DEGs were obtained. We also constructed regulatory networks of the key transcription factors with their predicted target genes (Figure S4A,B). From the regulatory network, we found that a small number of key TFs regulate a large number of target genes located on different chromosomes. These results show that the impact of aneuploidy on organisms is reflected in many aspects and affects many important biological processes. It also explains why aneuploidy seriously affects the growth and development of organisms at the individual level.

### 2.3. Identification and Enrichment Analysis of Differentially Expressed lncRNA Target Genes

**Figure 3:**
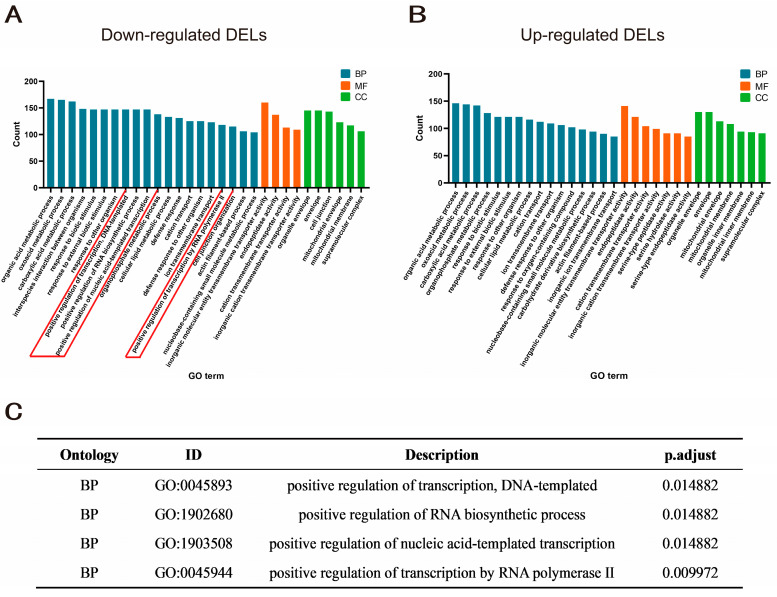
Enrichment of target genes for DELs. (**A**,**B**) GO enrichment results of target genes of down and upregulated DELs. The horizontal axis represents the enriched GO terms, the vertical axis represents the number of genes enriched for each GO term, and the three colors from left to right represent biological processes, molecular function and cellular composition, respectively. (**C**) The enrichment information for GO terms related to the positive regulation of transcription process.

lncRNAs play important roles in a variety of biological processes by regulating gene expression in various ways, which can be mainly divided into cis-regulation of neighboring genes and trans-regulation of distant protein-coding genes. Through colocalization and coexpression analyses, cis- and trans-target genes of the differentially expressed lncRNAs were identified. The results showed that for the 107 DELs, there were 2375 cis-regulated target genes and 2380 trans-regulated target genes in total (Table S1). To explore whether the regulation of the target genes by the DELs is genome-wide, we analyzed the density distribution of the cis-regulated and trans-regulated target genes across the chromosome (Figure S2B,C). The density distribution of target genes on chromosomes showed that the upregulated and downregulated target genes were widely distributed across the chromosomes, suggesting that aneuploidy has a wide range of effects on gene expression across the genome, and this broad effect was observed for both cis-regulated and trans-regulated target genes. Moreover, we found that the density of trans-regulated target genes was significantly higher than that of cis-regulated target genes, indicating that the regulatory effects of the DELs on their target genes were exerted mainly in trans. To further explore the role of these differentially expressed lncRNAs in the regulation of aneuploid gene expression, GO functional and KEGG pathway enrichment analyses of their target genes were performed. The GO enrichment analysis results showed that these target genes were not only involved in the metabolic processing of organic matter, material transmembrane transport, morphological structure and organ development, the oxidative stress response and other biological processes related to the metabolic development of aneuploidy, but also in the transcription process and positive regulation of RNA biosynthesis (Figure 3A–C). In addition, many genes were enriched in the supramolecular complex term in the cellular component ontology, and these enrichment analysis results indicated that lncRNAs may play an important role in the regulation of gene expression in aneuploidy.

Furthermore, by enrichment analysis of the target genes of the upregulated and downregulated DELs, we found that the enrichment results were roughly the same, but the GO terms related to transcriptional regulation appeared mainly in the enrichment results for the target genes of downregulated DELs and not in those for any of the target genes of upregulated DELs (Figure 3A,B). This indicates that in aneuploidy, lncRNAs play an important role in the regulation of growth and development processes, but the regulation of gene expression in aneuploidy involves primarily downregulated lncRNAs. To further explore TFs among the target genes of the downregulated DELs, enrichment analysis of TF-binding motifs in the sequences 5 kb upstream of the target genes of the downregulated DELs in metafemale *Drosophila* was performed. Motifs were screened according to the criterion of NES >3, and the 10 binding motifs with the highest scores are listed in Figure S5. By annotation with highconf, 17 enriched transcription factors were obtained. Among the 17 TFs, two TFs exhibited differential expression, where *Cnx14D* was upregulated and *ara* was downregulated (Figure S4C). The key transcription factors form a complex regulatory network with their predicted target genes, with a single transcription factor regulating a large number of genes on different chromosomes. This suggests that the regulation of target genes by lncRNAs occurs on a genome-wide scale (Figure S4C).

### 2.4. lncRNA-mRNA Interaction Network

To identify key lncRNAs related to the regulation of aneuploid gene expression, based on the enrichment results of the target genes, we screened the target genes in the GO terms related to transcriptional regulation (Figure 3C) and obtained the corresponding differentially expressed lncRNAs based on the target interaction relationship. The results were imported into Cytoscape software to construct the lncRNA-mRNA interaction network related to transcriptional regulation in aneuploidy. Among the 147 target genes related to aneuploidy, 41 were downregulated, 26 were upregulated, 7 were both transcriptionally upregulated and downregulated, and 73 had no significant differences in expression between metafemale individuals and normal diploid female individuals.

**Figure 4:**
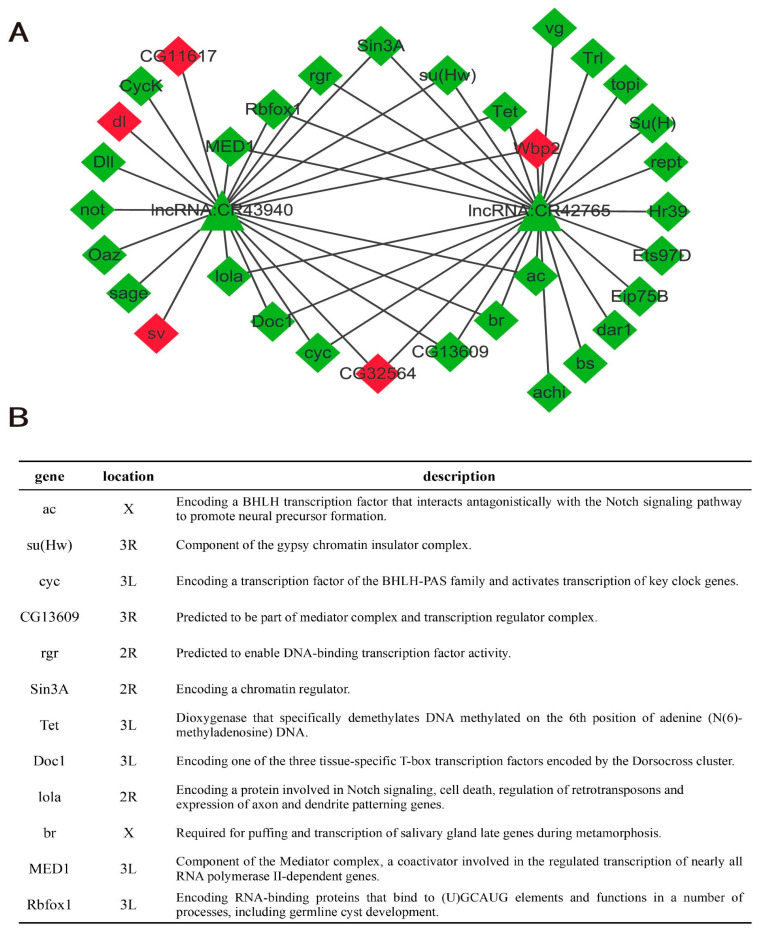
Hub lncRNAs and their target genes. (**A**) Subnetwork composed of lncRNAs with MCC values >30 and their target genes, where triangles indicate lncRNAs, diamonds indicate mRNAs, green indicates that the gene is downregulated in metafemale flies and red indicates upregulation. (**B**) Information on the common downregulated target genes of lncRNA CR43940 and lncRNA CR42765. Chromosomal location information and functional descriptions were obtained from the Flybase website (http://flybase.org/, accessed on 3 December 2021).

To construct an lncRNA-mRNA interaction network, we used the cytoHubba plug-in in Cytoscape to calculate the maximal clique centrality (MCC) value of each node in the network and sorted the nodes according to the MCC values from high to low. The results are shown in Table 1. Two lncRNAs with MCC > 30 (CR43940 and CR42765) were selected as hub lncRNAs in the network, and their corresponding target genes were screened to construct a subnetwork (Figure 4A). In the network, these two lncRNAs have many overlapping target genes and most of these target genes are downregulated (green diamonds) in metafemale *Drosophila* (Figure 4A). Among these downregulated overlapping target genes (Figure 4B), ac is a reported inverse dosage regulator whose overexpression on the X chromosome causes genes on autosomal chromosomes to exhibit an inverse dosage effect [34]. Sin3 is a large scaffolding protein that modifies chromatin structure and regulates gene expression by recruiting histone deacetylases and other transcription factors. The *Drosophila* genome encodes only one Sin3 protein, namely, Sin3A, which regulates almost 3% of *Drosophila* genes and has a profound impact on *Drosophila* gene expression [47]. MED1 is one of the components of the multiprotein mediator complex that mediates the expression of RNA polymerase II and transcription factors and is involved in regulating the expression of almost all genes that depend on RNA polymerase II transcription (Figure 4B). The changes in the expression of these genes may be one of the reasons that a large number of genes in metafemale *Drosophila* show dosage compensation or inverse dosage effects at the genome level. To understand the location relationship of the two hub genes and their cis-regulated target genes more clearly, we selected two representative target genes and mapped their locations on chromosomes 3L and 3R, respectively (Figure S6). The figure shows that the target genes are located very close to their corresponding hub lncRNAs on the chromosome, which makes their cis regulation by the hub lncRNAs very convenient. These results could provide valuable insight into the potential regulatory mechanism of these hub lncRNAs in metafemale *Drosophila.*

### 2.5. Validation of Candidate Inverse Dosage Modulators

**Figure 5:**
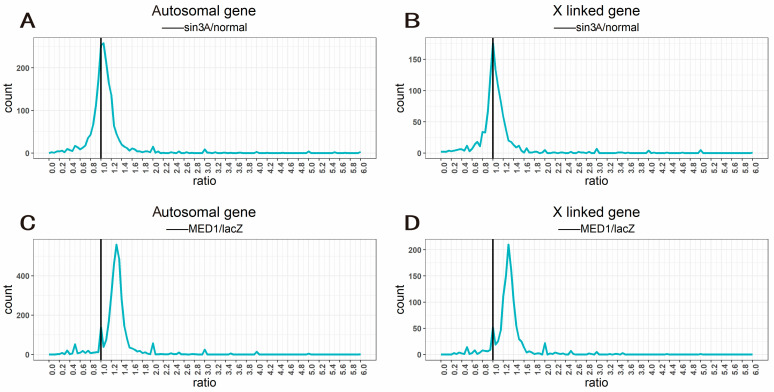
Ratio distribution of gene expression of candidate target gene mutants. (**A**,**B**) Ratio distribution of gene expression of *Sin3A* knockdown mutants, with wild type as the control group; all genes are divided into autosomal genes (**A**) and X linked genes (**B**) according to their positions on chromosomes. (**C**,**D**) Ratio distribution of gene expression of *MED1* knockdown mutants, with RNAi-mediated knockdown of exogenous *E. coli* LacZ gene as a control; all genes are divided into autosomal genes (**C**) and X linked genes (**D**) according to their positions on chromosomes. The ratio distribution map was plotted using the geom_freqpoly () function in the ggplot2 package. The horizontal axis represents the ratio of gene expression values compared to the control group, and the vertical axis represents the frequency ratios that fall into each bin of 0.05. The vertical black solid line represents the ratio 1.0 (no change).

To verify whether the above target genes are regulators of the inverse dosage effect, the expression matrix of mutants of these genes was searched in the GEO public database to explore whether their mutation affects expression levels in individuals at the genomic level. The GSE81221 dataset contains the gene expression profiles obtained after RNAi-mediated knockdown of 483 transcription factors in *Drosophila* S2R+ cells, including the profile obtained after *MED1* knockdown, while the GSE133064 dataset includes sequencing results of *Drosophila* with ovary-specific knockdown of *Sin3A*. The gene expression ratio distribution curve map was plotted to visualize the changes in expression levels in each mutant at the genomic level. For *Sin3A* knockdown mutants, the expression ratios of genes on the sex chromosomes were centered on a value slightly greater than 1.0, namely, a mild rightward shift occurred; the changes in autosomal genes, by contrast, were more pronounced, not only exhibiting a rightward shift, but also a value of the highest peak slightly greater than 1.0 (Figure 5A,B), indicating that knockdown of *Sin3A* resulted in a slight overall upregulation of gene expression. This phenomenon was more obvious in individuals with *MED1* knockdown mutations. Although the genes on autosomes and sex chromosomes had a low peak ratio of approximately 1.0, the expression ratio of most genes was centered around a value of 1.3, which was significantly increased (Figure 5C,D). Knockdown of genes involved in the positive regulation of transcriptional processes instead led to genome-wide upregulation of expression, similar to the effects of specific inverse dosage effect modulators reported previously [34], indicating that Sin3A and MED1 may also be potential inverse dosage modulators.

## 3. Discussion

The impact of aneuploidy on an individual is usually more severe than that of altered ploidy of the whole set of chromosomes [48]. This phenotypic effect of aneuploidy was observed as early as a century ago [10,12], but its molecular mechanisms are still poorly understood [9]. In the early days of the development of molecular genetics, the basis of this effect was thought to be the direct gene-dosage effect of the genes with changed copy numbers [8,49]. However, many subsequent studies showed that quite a few genes on the altered chromosomes produced a similar number of products compared with that in normal diploid individuals, that is, dosage compensation occurred [18,50,51]. In addition, some studies have proven that the expression of genes on the unaltered chromosomes in aneuploid individuals also changes, and the dominant effect is an inverse correlation with the dosage of the altered region, a phenomenon called the inverse dosage effect [18,19,21,28,50]. The results of our study show that the expression of genomic mRNA was affected by the dosage effect and inverse dosage effect resulting from the genomic imbalance and that genes simultaneously affected by the dosage effect and inverse dosage effect exhibit dosage compensation. In our study, the heatmap of the density distribution of differentially expressed genes on the chromosomes clearly shows that the effect of aneuploidy on gene expression is widespread (Figure 2E,F). The X chromosome of metafemale *Drosophila* showed dosage compensation, while the expression of genes on autosomes was affected by the inverse dosage effect (Figure 1A,B). To gain an in-depth understanding of the impact of aneuploidy on the regulation of genome-wide expression levels, we carried out enrichment analysis of genes corresponding to the differentially expressed mRNAs between metafemale *Drosophila* and normal diploid female *Drosophila* and found that many important biological processes were affected by the state of aneuploidy, for example, the metabolic processing of organic matter, the regulation of morphogenesis of the anatomical structure of *Drosophila* and the level of memory and cognition, which may explain why aneuploidy has such a serious impact on organisms. Among these effects, the effects of aneuploidy on mitochondrial function and metabolic pathways of some amino acids were extraordinarily significant (Figure 2G,H), probably because the gene dosage imbalance in aneuploidy induces proteotoxic stress and activates autophagy to maintain proteostasis [52]. In this process, saturation of autophagy leads to the impairment of the mitophagy in cells, and mitochondria themselves may also be affected by proteotoxic stress. Consequently, dysfunctional mitochondria accumulate intracellularly [53].

We also carried out an analysis of differentially expressed lncRNAs to explore the effects of lncRNAs on the regulation of gene expression in aneuploidy. By interacting with DNA, RNA, proteins and other molecules, lncRNAs are involved in the structural modification of chromatin, the recruitment of the transcription machinery and the processing and degradation of mRNA, regulating gene expression in cis or trans [37,38]. Considering that lncRNAs are also important transcriptional regulatory elements and have many target molecules (including transcription factors), we speculate that lncRNAs play an important role in the regulation of gene expression in aneuploidy. The results showed that aneuploidy status also had a genome-wide effect on lncRNA expression (Figure 2F), that lncRNAs were more sensitive to changes in dosage than mRNAs (Figure 1C,D), and that their peak ratio range on the gene expression ratio distribution plot was wider than that of mRNAs and showed a double inverse dosage effect on autosomes (Figure 1C) and overcompensation on the X chromosome (Figure 1D). As a rule, inverse dosage modulators are more sensitive to changes in dosage. For example, in previous studies, transcription factors and signal transduction components, which constitute the main group of inverse dosage modulators, showed a clear trans distribution, and the gene expression peak ratio in trisomy was less than 0.67 [8]. These results suggest that lncRNAs may be similar to some transcriptional regulators and signal transduction components and that they are also an important group of inverse dosage modulators.

Since lncRNAs mainly affect a series of biological processes by regulating the expression of target genes, we searched for and identified the over-represented target genes of the DELs between metafemale *Drosophila* and normal diploid female *Drosophila*. We found that the upregulated and downregulated target genes of the DELs were widely distributed across the chromosomes, suggesting that aneuploidy has a wide range of effects on gene expression across the genome, and this broad effect was observed for both cis-regulated and trans-regulated target genes (Figure S2B,C). Moreover, we found that the density of trans-regulated target genes was significantly higher than that of cis-regulated target genes, indicating that the regulatory effects of DELs on their target genes were mainly exerted in trans (Figure S2B,C). Interestingly, the target genes of the downregulated lncRNAs, but not those of the upregulated lncRNAs, were enriched in terms related to transcriptional regulation (Figure 3A,B), indicating that the regulation of gene expression in aneuploidy is mediated primarily by downregulated lncRNAs. This phenomenon may be explained by previous findings on transcription factors in trisomy, that is, trisomy reduces the expression of those unlinked lncRNAs, which in turn regulate the expression of target genes through cascade reactions [8].

The response of aneuploid gene expression to dosage depends on the regulatory network [30,54,55], and the increased connectivity of these genes to other members of the network makes them more sensitive to changes in dosage because there is potential for a titrating effect (i.e., useless intermediate complexes produced by overexpression) and a need for stabilization [56]. Therefore, we constructed an lncRNA-mRNA interaction network and searched for nodes with high correlation in the network (lncRNA CR43940 and lncRNA CR42765) to identify hub genes (Table 1). Following the above line of reasoning, these nodes should be key components in the regulatory network of aneuploid gene expression. Subsequent research results confirmed this hypothesis, and two of the downstream target genes were potential inverse dosage modulators. However, there is not much information on the functional annotation and description of these two lncRNAs, and further research is needed to explore their specific roles in the regulatory network of aneuploid gene expression.

By studying the regulators of the white gene in *Drosophila*, it was found that the regulatory mechanism of dosage change in aneuploidy can be narrowed down to the role of single genes [31,32] called inverse dosage modulators. Their functions are often related to expression regulation, and most of these genes are components of macromolecular complexes [29,57]. In this study, we also focused on certain genes related to transcriptional regulation and found several possible inverse dosage modulators. Among them, ac (achaete) is a BHLH transcription factor that usually forms a complex (AS-C) with sc (scute), which promotes the formation of neural precursors in metazoans [58,59]. In a previous study, overexpression of ac produced an effect similar to that of aneuploidy, causing genes on autosomes to exhibit an inverse dosage effect, indicating that ac is an inverse dosage modulator [34]. Sin3A is a large scaffold protein that modifies chromatin structure by recruiting histone deacetylases and other transcription factors, thereby regulating gene expression [47]. It is a genome-wide transcriptional regulator with the ability to positively or negatively regulate the expression of various genes and thus participate in the regulation of various biological processes, including cell proliferation, differentiation, apoptosis and cell cycle progression, and it is related to various signaling pathways, such as the Hippo and JNK pathways [60,61]. MED1 is one of the components of the multiprotein mediator complex, which mediates the action of gene-specific transcription factors at enhancers and the transcriptional mechanism of RNA polymerase II (RNAPII) at promoters. It also interacts with other factors involved in transcription, chromatin regulation and mRNA processing, and is the node of RNAPII-mediated transcription [62]. As a coactivator, MED1 is involved in almost all RNAPII-dependent transcriptional regulatory processes [63]. Our results showed that knockdown of *Sin3A* and *MED1* shifted the peak on the gene expression ratio distribution plot to the right, indicating upregulation of gene expression (Figure 5), an effect that may be explained by the model of the gene balance hypothesis [64]. However, it should be noted that although mutations in *Sin3A* and *MED1* can mimic the regulation of aneuploid gene expression, the magnitude of this regulation is relatively small, especially for *Sin3A* (Figure 5A,B). To a certain extent, this also shows that the regulation of gene expression by aneuploidy is actually the result of a series of dosage-sensitive regulatory factors acting as members of macromolecular complexes to form a complex gene expression regulatory network [34].

The effects of aneuploidy on the genome have not only been demonstrated in *Drosophila*; similar results have been found in other species, such as maize and humans [50,65,66,67]. The results of previously reported research on DS show that most of the chromosome 21 transcripts are compensated for the gene-dosage effect [65]. In addition, by mimicking sex-chromosome dosage (SCD) effects in lymphoblastoid cell lines, scientists found that the downregulated expression of X-linked genes with increasing X-chromosome number and these effects of SCD occurred broadly across the genome, with potential implications for human phenotypic variation [66]. Furthermore, research on human sex-chromosome aneuploidy diseases, Klinefelter syndrome (KS) and Turner syndrome(TS), shows that there are 94 DEGs that overlap between TS and female and KS and male comparisons, indicating the existence of common molecular mechanisms for gene regulation in TS and KS that transmit the gene dosage changes to the transcriptome [67]. These results all support our research results to a certain extent, and the study of *Drosophila* aneuploidy is of great significance to the investigation of the mechanism of human aneuploidy disease.

The severe phenotypic effects of aneuploidy on individuals may originate from genomic imbalance [56,68]. Early explanations of genome balance were mainly based on two different viewpoints: the balance of enzymes/metabolism and the balance of gene expression regulation, but subsequent accumulating evidence suggested that most of the balance mechanisms may reflect some form of balance of gene expression regulation [68]. With regard to the influence of aneuploidy on the regulation of gene expression, some current studies have provided further understanding. For example, the effect of aneuploidy is progressive: the more severe the aneuploidy is, the more pronounced the effect [30,69]. Genes in different functional groups showed different responses to aneuploidy [8,69], transcription factors and signal transduction components were more obviously regulated by inverse dosage effects, and the responses of transcription factors and their target genes showed significant inconsistency [8]. Considering these findings and the observation that the condition of aneuploidy likely reflects a disturbed balance of regulated genes in the genome, the gene regulation mechanism may be explained by the gene balance hypothesis [56]. The gene balance hypothesis states that changes in the stoichiometry of multisubunit complex components affect the assembly kinetics of the complex, which in turn affects the number of functional products and gene expression patterns, ultimately affecting the phenotype and fitness of individuals [5]. Subsequent studies have also shown that most of the components of these multisubunit complexes are transcription factors, signal transduction components and chromatin modifiers, consistent with the findings listed above [33]. Although this gene regulatory mechanism involves numerous complex processes and has not been elucidated in detail, studies on quantitative traits, haploinsufficiency, evolutionary genomics and copy number variation (CNV) have all demonstrated that dosage changes in macromolecular complex components may be related to the regulation of gene expression in aneuploidy [5,56,57,68,70,71,72]. However, the regulation of genomic imbalance in aneuploidy at the level of genomic expression may be more complex than we determined; some studies have shown that in addition to lncRNAs, microRNAs and TEs are also involved [73,74,75]. The regulatory networks and specific molecular mechanisms remain to be further explored. 

## 4. Materials and Methods

### 4.1. Drosophila Stocks and Crosses

The *Drosophila* strains used in the study were Canton S wild type and C (1) DX, ywf/winscy, which were cultured on cornmeal sucrose medium at 25 °C. The metafemale *Drosophila* samples were obtained from crosses of ywf/winscy females with Canton S males. The adult flies in the culture flasks were emptied and cultured at 25 °C, and fruit flies newly emerged within 10 h were collected. Since it is difficult for metafemale *Drosophila* to survive to the adult stage, screening begins at the third-instar larval stage of the progeny. Three types of viable third-instar larvae were obtained after crossing: metafemale larvae with black mouthparts (C(1) DX^y−^ X^y+^), male larvae with black mouthparts (X^y+^ Y) and female larvae with yellow mouthparts (C(1) DX^y−^ Y). Metafemale larvae with black mouthparts can be screened according to sex and mouthpart color (Figure S7). At the same time, the third-instar larvae of Canton S wild-type *Drosophila* were selected as controls. The collected samples were placed in a −80 °C freezer for subsequent experiments.

### 4.2. RNA Extraction and Sequencing

RNA was extracted using TRIzol Reagent obtained from Invitrogen (Thermo Fisher Scientific, Waltham, MA, USA) and processed using a *DNase I, RNase-Free kit* obtained from Thermo Scientific (Thermo Fisher Scientific, USA). The collected RNA was sent to the company for sequencing. Briefly, quality detection and concentration measurement were performed on RNA samples, then an RNA-Seq library was constructed. Illumina PE150 (paired-end 150 nt) high-throughput paired-end sequencing was performed according to the effective concentration of the library.

### 4.3. RNA Sequencing Analysis

RNA-Seq sequencing data were analyzed using Hisat2 software (version 2.0.5) [76]. For lncRNAs, Cuffmerge software (version 2.2.1) [77] was used to trim, filter and screen database annotation lncRNAs for subsequent analysis. The *Drosophila* reference genome and genome annotation information used in this process were downloaded from the Ensembl database (reference genome: ftp://ftp.ensembl.org/pub/release-102/fasta/drosophila_melanogaster/dna/; genome annotation file: ftp://ftp.ensembl.org/pub/release-102/gtf/drosophila_melanogaster/, accessed on 10 March 2021).

Stringtie software (version 1.3.3) was used to splice the reads matched to the genome into transcripts and quantify them [78]. After obtaining the read count value expressed by each gene, FPKM (fragments per kilobase of transcript sequence per millions base pairs sequenced) values were calculated. These values simultaneously correct the effects of sequencing depth and gene length on fragment counts, and the method is currently the one most commonly used for estimating gene expression levels. Principal component analysis (PCA), boxplot and clustered heatmap of sample distances were plotted to demonstrate the good repeatability (Figure S8).

### 4.4. Ratio Distribution

The data from RNA sequencing were normalized to calculate CPMs (counts per million). Using the CPM of each gene in normal diploid female *Drosophila* as a control, the ratio of the experimental group to the control group was calculated, and the ratio distribution plots was generated using the ggplot2 (version 3.3.5) [79] package in the R program. The sequencing data used to draw the mutant ratio distribution plots were sourced from the GEO database (https://www.ncbi.nlm.nih.gov/geo/, accessed on 27 January 2022), and the sequencing data for the *Sin3A* knockdown mutation came from GSE133064, while the sequencing data for the *MED1* knockdown mutation came from GSE81221.

### 4.5. Differential Expression Analysis

Differential gene expression analysis was performed using the DESeq2 package (version 1.34.0) [80], resulting in log2FoldChange values (log2FC), *p*-values (Wald test), and adjusted *p*-values (padjs). To avoid the large dispersion of log2FC values at low read counts, the log2FC values were shrunk using the apeglm package (version 1.16.0) [81]. Differentially expressed genes were screened, with |log2FC| > 1 and padj < 0.05 as criteria, where log2FC > 1 and log2FC < −1 represented upregulated and downregulated genes, respectively. The heatmap of differential genes was plotted using the pheatmap package (version 1.0.12), and the heat map of the density distribution of differential genes on chromosomes was drawn using the RIdeogram package (version 0.2.2) [82].

### 4.6. Differential lncRNA Target Gene Prediction

The regulation of lncRNAs on target genes is mainly divided into cis and trans. The prediction of cis-target genes is mainly based on the positional relationship (co-location) of lncRNA and target genes in the genome, and they are determined by finding genes within 10kb upstream and downstream of lncRNAs; trans-target genes are mainly determined according to the expression correlation (co-expression) of lncRNAs and mRNAs. By calculating the Pearson correlation coefficient (PCC) between the differential lncRNA and mRNA of each sample and selecting the lncRNA-mRNA pair whose absolute correlation coefficient value is greater than 0.95 (|PCC| > 0.95) and whose *p*-value < 0.01, the trans-target genes of differential lncRNAs can be obtained.

### 4.7. Target Gene Enrichment Analysis

The clusterProfiler package (version 4.2.0) [83] was used to perform Gene Ontology (GO) enrichment and KEGG Pathway enrichment analysis on genes with differential mRNAs and target genes of differential lncRNAs. The GO annotation information was sourced from “org.Dm.eg.db” (version 3.14.0), and the KEGG database was obtained from online data (https://www.genome.jp/kegg-bin/show_organism?org=dme, accessed on 15 July 2021). For the target genes of differential lncRNAs, GO terms with padj values less than 0.05 were screened and classified into one of three categories: Molecular Function (MF), Biological Process (BP) or Cellular Component (CC), then plotted in a GO enrichment histogram.

### 4.8. Transcription Factor Analysis

Transcription factor enrichment analysis was performed using RcisTarget (version 1.10.0) in R. The over-expressed TF-binding motifs of the upstream 5 kb sequence of the selected downregulated genes were analyzed using the database provided. Then, the possible regulatory transcription factors were searched based on these enriched motifs and screened according to their expressions [84]. The circular diagrams of regulatory networks for candidate transcription factors were plotted using Circos (version 0.69.8) [85].

### 4.9. lncRNA-mRNA Interaction Network

Target genes annotated with transcriptional regulation and chromatin modification functions were screened, and corresponding differential lncRNAs were obtained according to the target interaction relationship. The results were imported into Cytoscape software (version 3.8.2) [86] to construct an lncRNA-mRNA interaction network, and the cytoHubba (version 0.1) [87] plugin was used to calculate the hub genes in the interaction network. Since the MCC method has good performance in network prediction [87], the calculation results were sorted according to the MCC values, and the lncRNAs with higher MCC values were selected as the key lncRNAs.

## 5. Conclusions

In this study, transcriptome data of metafemale *Drosophila* and normal diploid female *Drosophila* were analyzed, and the phenomenon of dosage compensation and inverse dosage effects of mRNAs and lncRNAs in aneuploidy on a genome-wide scale were observed. Functional annotation of differentially expressed genes explains why aneuploidy causes severe phenotypic effects at the molecular and cellular levels. To search for key factors in the regulatory network of gene expression in aneuploidy, we constructed an lncRNA-mRNA interaction network and verified the role of two potential inverse dosage modulators, and found that their knockdown resulted in a genome-wide upregulation of gene expression, indicating that mutations in a single regulatory factor can mimic the effects of aneuploidy on gene expression, producing inverse dosage effects. Most importantly, we found that lncRNAs are more sensitive to dosage changes than mRNAs and that aneuploidy status affects lncRNA expression, which in turn affects downstream gene expression through a cascade, including expression of transcription factors. We also discovered two key lncRNAs, although their specific roles remain to be explored. Most previous studies on aneuploidy only focused on the role of protein-coding genes; this study, however, revealed that noncoding RNAs also play a role in the regulation of gene expression in aneuploidy by analyzing the expression of lncRNAs in metafemale *Drosophila*. This will help scientists to further understand the expression regulation mechanism of aneuploidy.

## Figures and Tables

**Table 1:** The calculation results for the lncRNA-mRNA interaction network.

Node_Name	MCC	DMNC	MNC	Degree	EPC	Bottleneck	Eccentricity	Closeness	Radiality	Betweenness	Stress
lncRNA:CR43940	31	0	1	32	50.165	4	0.19721	96.48333	6.27612	3619.788	202,948
lncRNA:CR42765	30	0	1	30	49.031	4	0.19721	94.9	6.21069	3721.355	213,714
lncRNA:CR46258	29	0	1	29	49.566	3	0.19721	94.11667	6.20134	2571.495	165,596
br	25	0	1	25	44.288	4	0.16434	96.13333	6.24808	5976.691	263,602
lncRNA:CR45232	25	0	1	25	46.97	6	0.19721	90.03333	6.08919	2361.111	173,502
lncRNA:CR44948	23	0	1	23	45.544	6	0.19721	86.81667	5.99572	1835.18	130,014
lncRNA:CR45170	23	0	1	23	45.698	1	0.19721	90.51667	6.17331	2923.464	174,554
lncRNA:CR43651	22	0	1	22	46.481	2	0.19721	88.55	6.08919	1555.737	120,006
lncRNA:CR45972	21	0	1	21	44.079	17	0.19721	87.28333	6.07049	2236.299	136,518
Wbp2	20	0	1	20	44.254	52	0.16434	88.28333	6.07049	2891.457	144,714

## Data Availability

The sequencing data have been deposited in the Gene Expression 695 Omnibus (GEO) database (https://www.ncbi.nlm.nih.gov/geo/, accession no. GSE224569, accessed on 7 February 2023).

## Supplementary Materials

The supporting information can be downloaded at: https://www.mdpi.com/article/10.3390/ijms24098381/s1.
