# Supplementary material for: Genome-Wide Expression Analysis of Long Noncoding RNAs and Their Target Genes in Metafemale Drosophila
Source: Int J Mol Sci. 2023 May 6;24(9):8381. doi: 10.3390/ijms24098381 (PMC10179461; doi:10.3390/ijms24098381)
Supplement: Supplementary file 1 [file ijms-24-08381-s001.zip › ijms-2298790-supplementary.pdf]

International Journal of
Molecular Sciences

MDPI

supplementary information

Genome-wide expression analysis of long noncoding RNAs and their target genes in metafemale *Drosophila*

Xinyu Liu, Ran Yan, Haosheng Liu, Shuai Zhang, Ruixue Wang, Bowen Zhang and Lin Sun

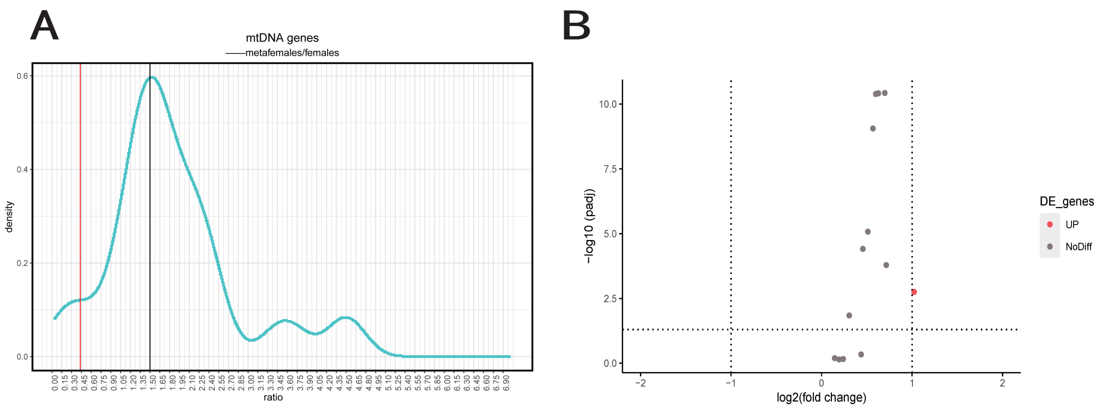

Figure S1 (A) Ratio distributions of mitochondrial gene expression in metafemale (XXX) compared with normal diploid. The vertical red solid line represents the ratio 0.44 (the ratio of double inverse dosage effects $(2/3)^2$), the vertical black solid line shows the ratio 1.50 (the ratio of gene dosage effects (3/2)). The frequencies were plotted in bins of 0.05. (B) Volcano map of DEGs of mitochondrial gene in metafemale (XXX) compared with normal diploid. DEGs are defined as adjusted p-value < 0.05 and | fold change | >1.00.

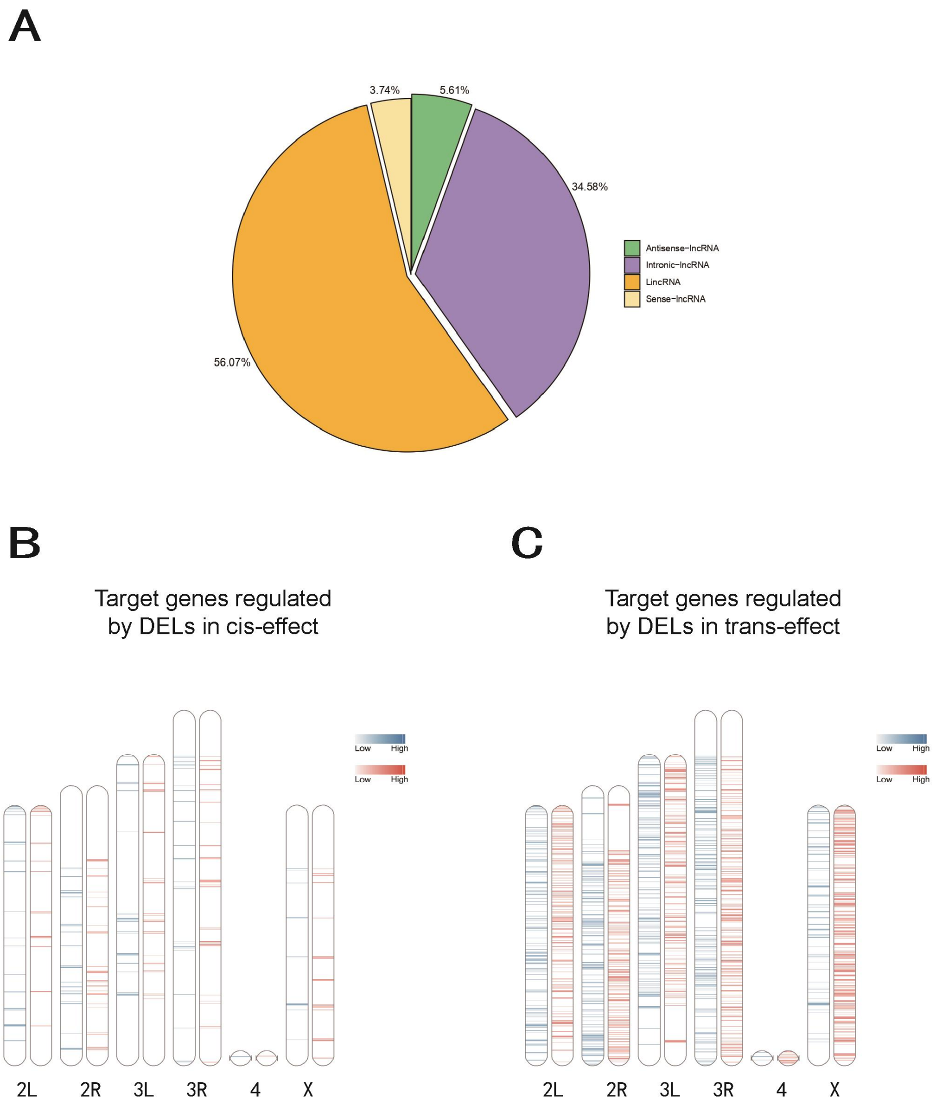

Figure S2 proportion of four kinds of DELs and density heatmaps of the distribution of target genes of DELs along chromosomes in metafemale Drosophila. (A) Classification criteria for lncRNAs are based on their position in the genome relative to protein-coding genes, including intergenic lncRNAs (LincRNAs), intronic lncRNAs, sense lncRNAs and antisense lncRNAs. (B) Target genes regulated by DELs in cis-effect. (C) Target genes regulated by DELs in trans-effect. The red bands represent up-regulated genes, and the blue bands represent down-regulated genes, respectively. The darker colors show a greater density of the genes.

A

	logo	motif	NES	AUC	TF_highConf	nEnrGenes
1	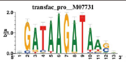	transfac_pro__M07731	5.96	0.06	pnr	680
2	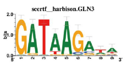	scertf__harbison.GLN3	5.81	0.06	GATAd; GATAe; grn; pnr; srp	613
3	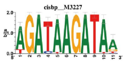	cisbp__M3227	5.77	0.06	-	691
4	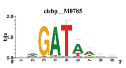	cisbp__M0785	5.72	0.06	pnr	659
5	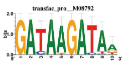	transfac_pro__M08792	5.7	0.06	CG10348; ham	648
6	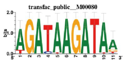	transfac_public__M00080	5.57	0.05	-	657
7	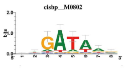	cisbp__M0802	5.56	0.05	GATAd; GATAe; grn; pnr; srp	638
8	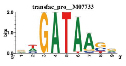	transfac_pro__M07733	5.55	0.05	srp	628
9	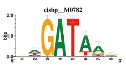	cisbp__M0782	5.49	0.05	pnr	648
10	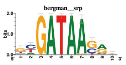	bergman__srp	5.47	0.05	srp	660

B

	logo	motif	NES	AUC	TF_highConf	nEnrGenes
1	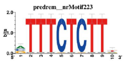	predrem__nrMotif223	4.48	0.07	-	92
2	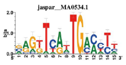	jaspar__MA0534.1	4.34	0.07	usp	72
3	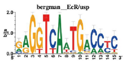	bergman__EcR_usp	4.12	0.07	EcR; usp	423
4	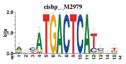	cisbp__M2979	3.84	0.07	cnc	87
5	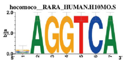	hocomoco__RARA_HUMAN.H10MO.S	3.82	0.07	-	102
6	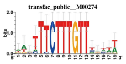	transfac_public__M00274	3.82	0.07	-	315
7	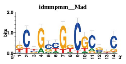	idmmpmm__Mad	3.8	0.07	Mad	110
8	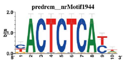	predrem__nrMotif1944	3.77	0.07	-	137
9	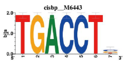	cisbp__M6443	3.76	0.07	-	48
10	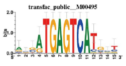	transfac_public__M00495	3.75	0.07	cnc	119

Figure S3 Enrichment analysis of transcription factors for up-regulated and down-regulated genes in metafemale *Drosophila*. The analysis is performed in up-regulated(A) and down-regulated (B), respectively. The over-represented motifs are ordered by normalized enrichment score (NES) with a threshold of 3, and the 10 binding motifs with the highest scores are listed.

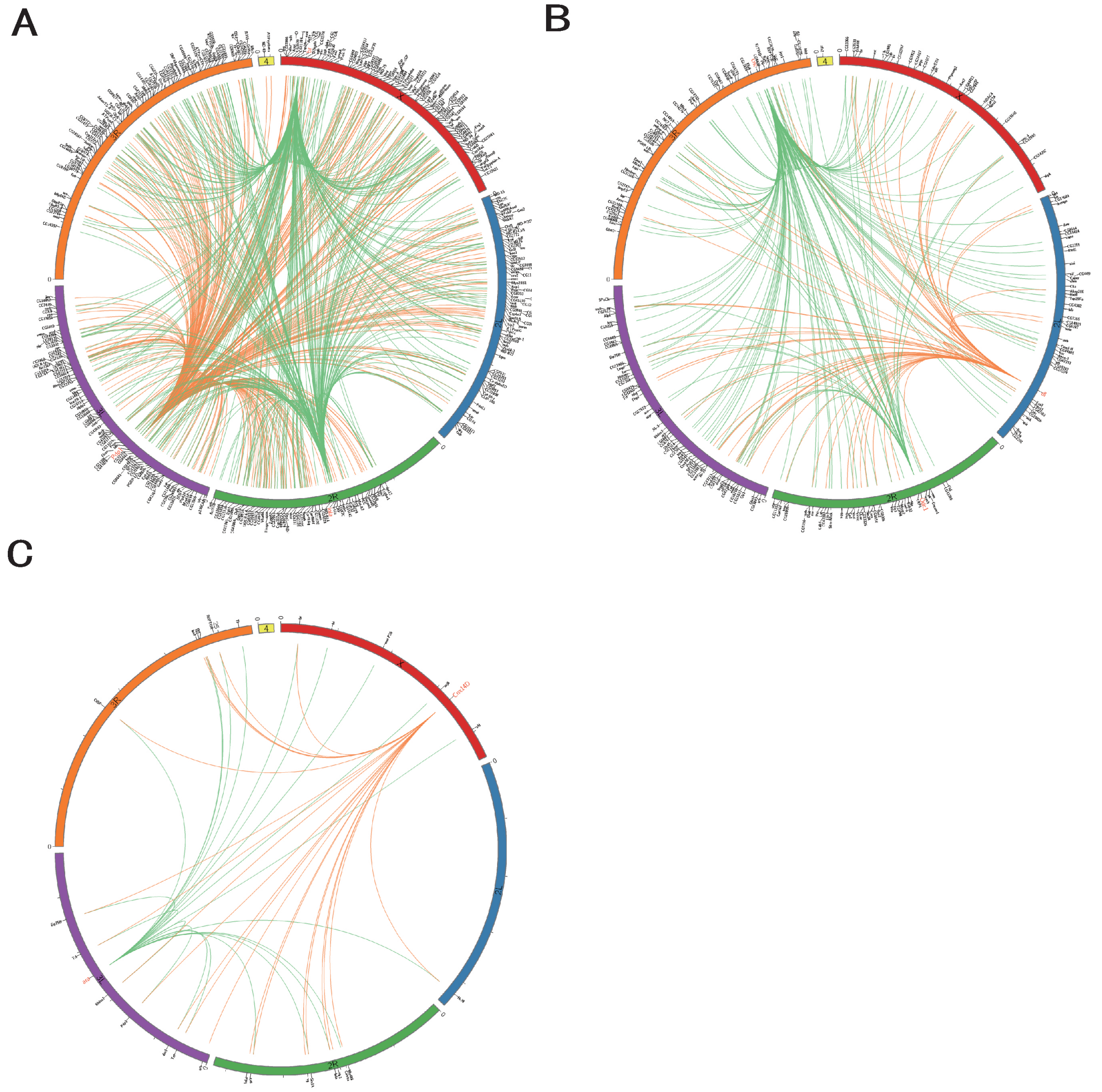

Figure S4 Modulation of gene expression by candidate transcription factors. (A–C) The circular plots show the target gene networks of candidate TFs in up-regulated DEGs (A), down-regulated DEGs (B) and down-regulated lncRNAs(C). The green lines indicate that the expression of this TF is down-regulated in metafemale *Drosophila*, and the orange lines indicate that the expression of this TF is up-regulated in metafemale *Drosophila*. Candidate TFs are labeled in red. Target genes are partially listed on the periphery of the chromosomes.

Table S1 Statistics of cis- and trans-target genes for DELs

	Up-regulated	Down-regulated	Targets with both up- and down-regulated transcripts	Unchanged	Sum
Cis-targets	264	163	19	1929	2375
Trans-targets	1455	818	107	0	2380

A

	logo	motif	NES	AUC	TF_highConf	nEnrGenes
1	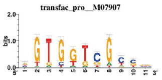	transfac_pro__M07907	3.82	0.26	-	16
2	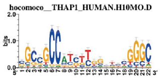	hocomoco__THAP1_HUMAN.H10MO.D	3.71	0.25	CG10431	23
3	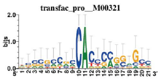	transfac_pro__M00321	3.60	0.25	-	26
4	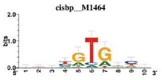	cisbp__M1464	3.56	0.25	-	20
5	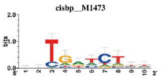	cisbp__M1473	3.52	0.24	-	14
6	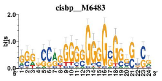	cisbp__M6483	3.50	0.24	Spps	26
7	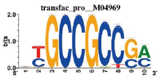	transfac_pro__M04969	3.47	0.24	-	21
8	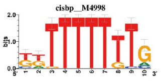	cisbp__M4998	3.45	0.24	hb	10
9	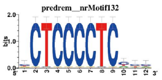	predrem__nrMotif132	3.44	0.24	-	22
10	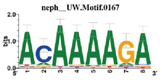	neph__UW.Motif.0167	3.44	0.24	-	25

Figure S5 Enrichment analysis of transcription factors for target genes of down-regulated lncRNAs in metafemale *Drosophila*. The over-represented motifs are ordered by normalized enrichment score (NES) with a threshold of 3, and the 10 binding motifs with the highest scores are listed.

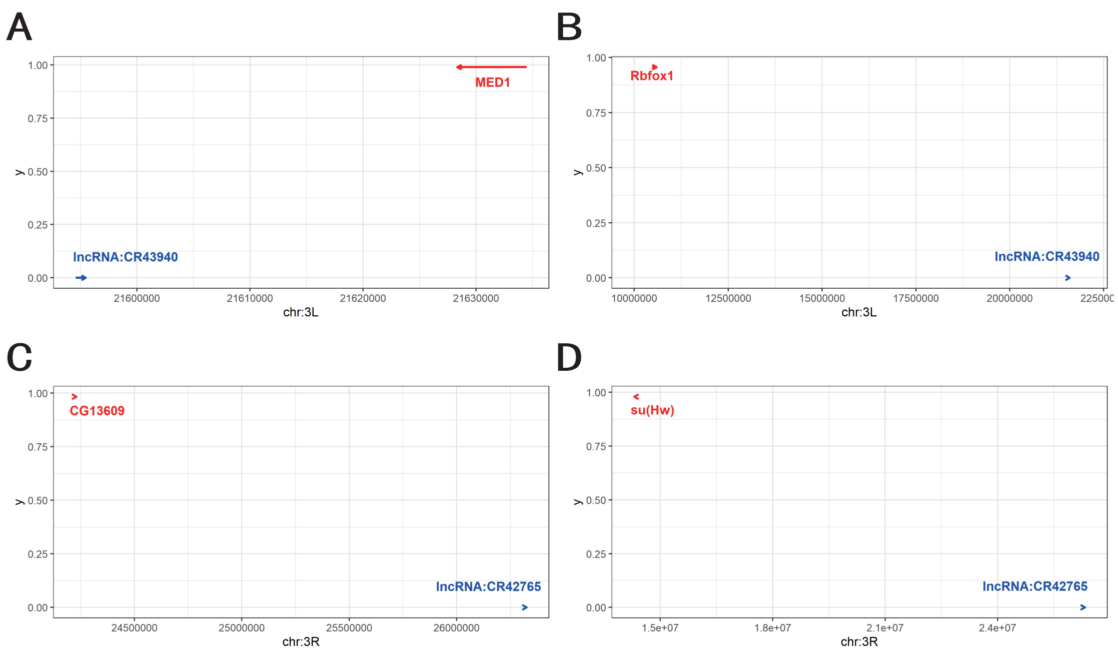

Figure S6 Representative lncRNAs and the positions of their cis genes in the chromosomes. The x axis represents the chromosome genome position, and the y axis represents the correlation coefficient of the lncRNAs and the cis genes, with a greater correlation coefficient corresponding to a higher position. The red arrow indicates the genome location of the encoding genes regulated by lncRNAs. The blue arrow indicates the location of lncRNA.

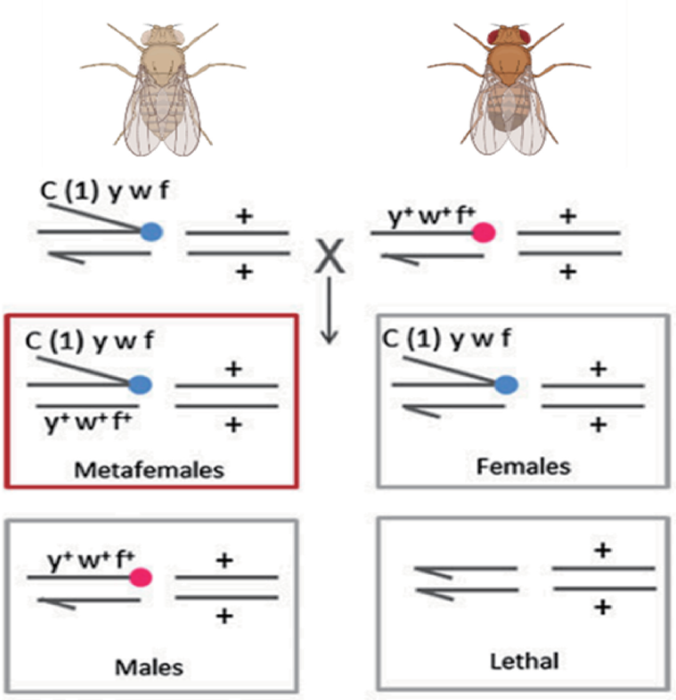

Figure S7 Genetic crosses to generate aneuploidy Drosophila. Cross of C(1)DX, ywf/winscy females with Canton S males to obtain metafemale larvae. The selected offspring genotype is shown in the red box.

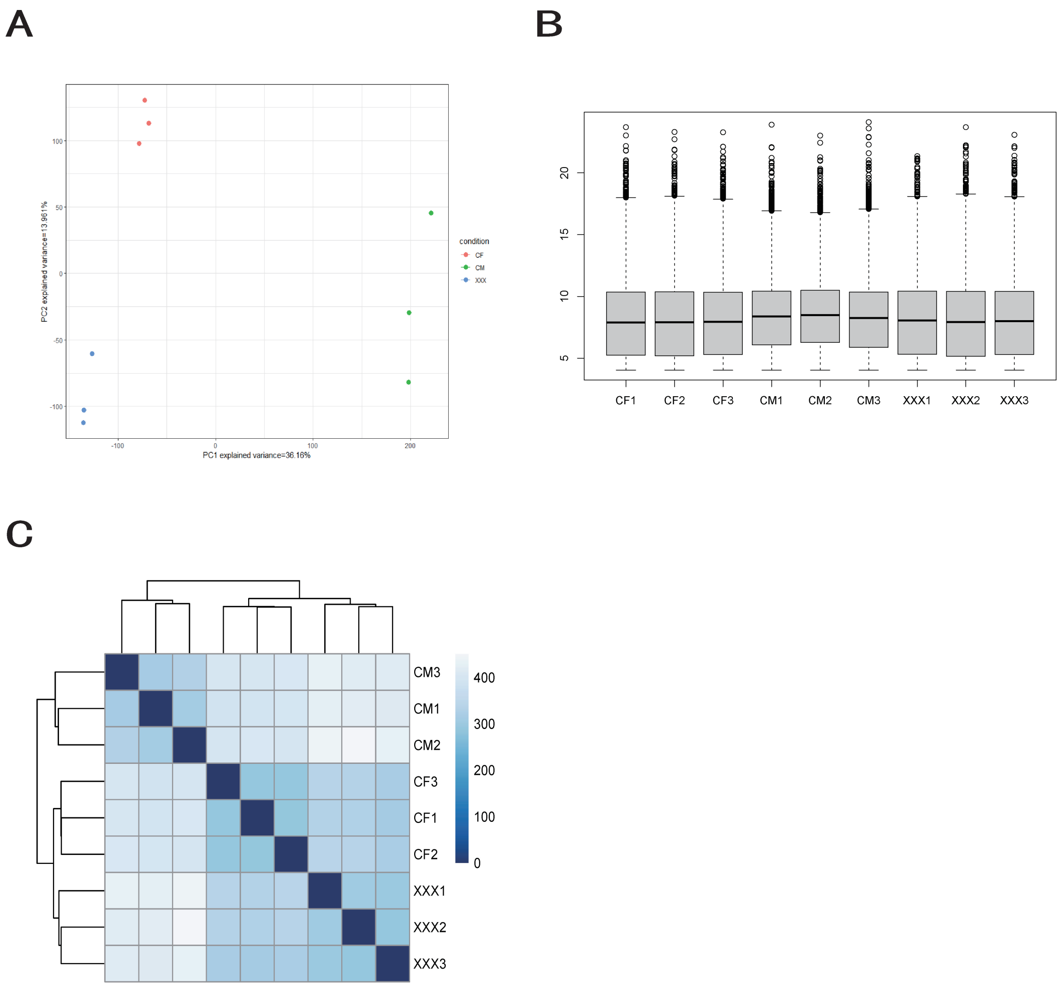

Figure S8 Principal component analysis (PCA), boxplot and clustered heatmap of sample distances of male, female and metafemale samples. R package DESeq2 was used for differential expression analysis of the expression matrix, and boxplot and PCA were drawn after vst standardization. The PCA diagram was drawn by R package ggplot2. The clustered heatmap of sample distances was measured using Euclidean distance, and the heatmap was drawn by R package pheatmap.
